# Structural architecture of the Middle Niger Basin, Nigeria based on aeromagnetic data: An example of a non-volcanic basin

**DOI:** 10.1016/j.heliyon.2021.e07205

**Published:** 2021-06-04

**Authors:** Naheem Banji Salawu, Silas Sunday Dada, Julius Ogunmola Fatoba, Olusola Johnson Ojo, Leke Sunday Adebiyi, Abayomi John Sunday, Toyin Yusuf Abdulraheem

**Affiliations:** a BS Geophysical and Consultancy Ltd., Ilorin, Nigeria; b Department of Geology and Mineral Sciences, Al-Hikmah University, Ilorin, Nigeria; c Department of Geophysics, Federal University Oye-Ekiti, Oye-Ekiti, Nigeria; d Department of Geology, Federal University Oye-Ekiti, Oye-Ekiti, Nigeria; e Department of Physical Sciences, Landmark University, Omu-Aran, Nigeria; f Department of Science Laboratory Technology, Kwara State Polytechnic, Ilorin, Nigeria

**Keywords:** Middle Niger Basin, Rift, Aeromagnetic data, Shear zone

## Abstract

The Middle Niger Basin is located within the west-central half of Nigeria, as a NW-SE trending Campano-Maastrichtian depo-centre which extends from the southern end of the Sokoto Basin west of Kainji reservoir southwestwards to the convergence of the Benue and Niger Rivers at Lokoja. The aeromagnetic anomaly data of the Middle Niger Basin was interpreted to characterize the structural architecture and depth to the magnetic basement of the basin. This is to expand the current knowledge of the region. The analysis of the data has been facilitated by the application of derivative and source parameter imaging techniques. The results from the application of total gradient to the aeromagnetic data provided here signify a rift origin of the basin and NW-SE trending fault systems in the surrounding basement complex terrain. The absence of magnetic highs on the first vertical derivative of the reduced-to-pole aeromagnetic data reveals lack of volcanic rocks within the sedimentary layers of the basin. Additionally, the consistent NW-SE trending source parameter imaging depth solutions within the basin confirm the internal geometry and NW-SE orientation of the basin with sediments not more than 1100 m thick.

## Highlights

• Boundaries of basement areas with the basin are marked by systems of lineaments.
• Delineated lineaments offer target areas for follow-up mineral investigation.
• FVD map reveals possible lack of volcanic rocks within the basin.
• Internal geometry of the basin is revealed based on SPI depth solutions.

## 1 Introduction

**Figure 1:**
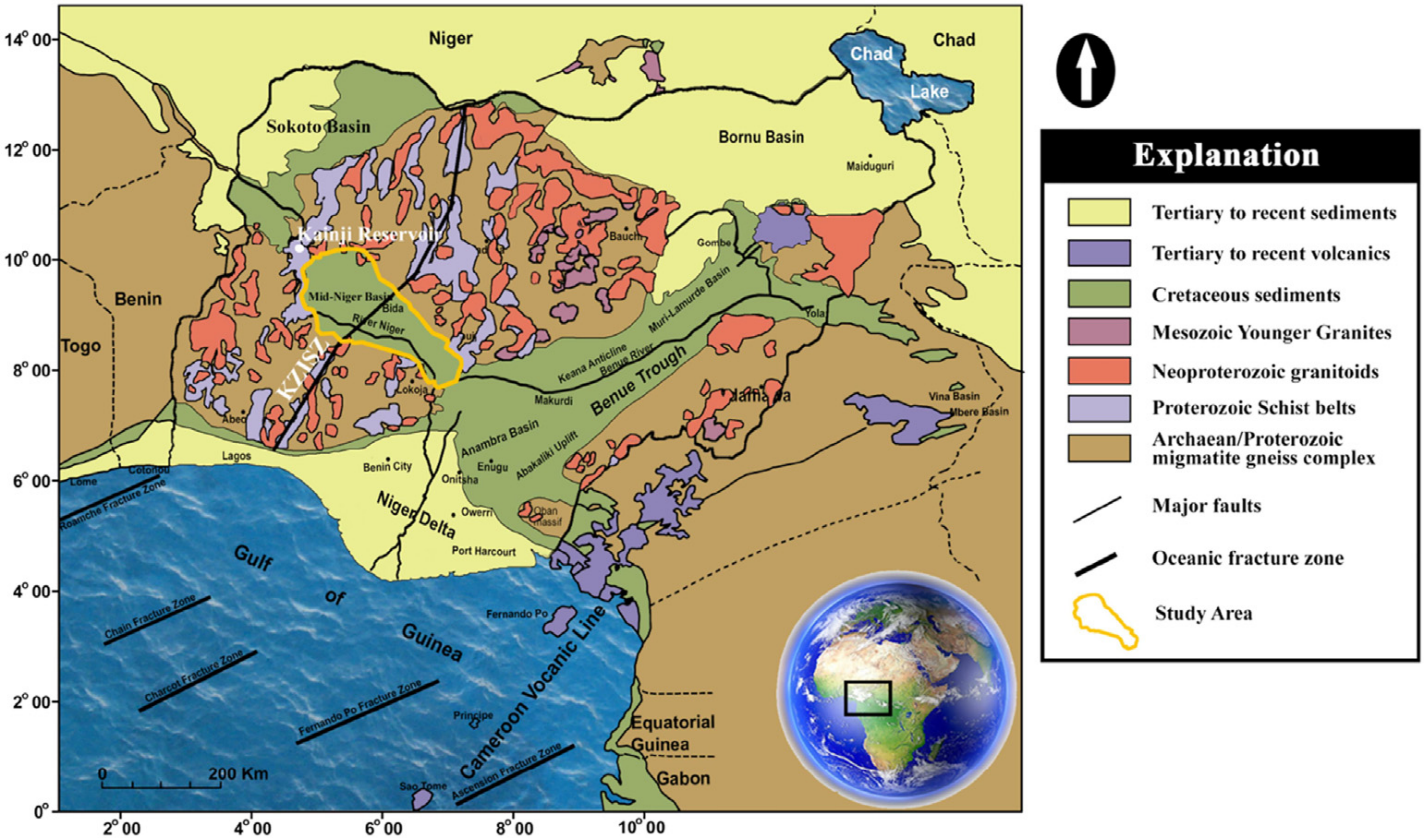
Simplified geological map of Nigeria and its surrounding areas showing the location of the Middle Niger Basin, which is indicated with a yellow polygon on the map. (Adapted from Salawu et al., 2021). The Abbreviation used: KZISZ - Kalangai-Zungeru-Ifewara shear zone. Reprinted by permission from [the Licensor]: [Springer Nature] [Geomechanics and Geophysics for Geo-Energy and Geo-Resources] [Structural geometry of Ikogosi warm spring, southwestern Nigeria: evidence from aeromagnetic and remote sensing interpretation, Salawu et al., (2021), [COPYRIGHT] (2021). Journal website: https://www.springer.com/journal/40948.

The Middle Niger Basin, which extends northwesterly from the convergence of the River Niger and River Benue at the Lokoja/Dekina axis, is flanked on both sides by the basement complex of northwestern and southwestern Nigeria (Figure 1). It equally separates the basin from the early Cretaceous to late Jurassic Illo and Gundumi Formations of the Sokoto Basin by a narrow geologic formation considered to be crystalline basement around the Kainji reservoir area (Adeleye and Dessauvagie, 1972; Kogbe 1989; Rahaman et al., 2018). The Middle Niger basin is fairly elliptical with an orientation that is virtually perpendicular to the Benue Trough in Nigeria. The basin is roughly 350 km long with a width between 75 km and 150 km (Zaborski 1998). The Middle Niger Basin is different from other basins in Nigerian because of the absence of carbonate rock. The regional structural evolution of the basin has been a subject of debate for long and satisfactory clarification about its tectono-stratigraphic evolution is still in contention (Rahaman et al., 2018).

Several researchers that investigated the Middle Niger Basin have reported contradictory thicknesses for the sedimentary pile. A possible maximum depth value of Middle Niger Basin sediments has been estimated to be generally around 1000 m (Kogbe et al., 1983). Ojo (1990), based on aeromagnetic investigation, reported basic intrusive rocks at depths between 4000 m–6000 m well below the base of the sedimentary formations of the Middle Niger Basin, which he suggested to be not more than 2000 m thick. Udensi and Osazuwa (2004) carried out magnetic investigation of the central and southern portion of the Middle Niger Basin using spectral analysis. Their results reported a sedimentary thickness of about 3500 m within the investigated segment of the basin. Megwara and Udensi (2014) used aeromagnetic data to investigate the southern portion of the Middle Niger Basin and concluded that the depths to the magnetic sources along established aeromagnetic data profiles range from 10 to 510 m with an average depth value of 128 m. Salawu et al. (2020) integrated remote sensing and aeromagnetic methods for the investigation of structural features and possible depth to magnetic sources of the northern segment of the Middle Niger Basin with a view to unravel the sedimentary thickness and lineaments of the studied region. The results of their investigations revealed a maximum sedimentary thickness of 950 m within the northern portion of the basin and also showed that the boundaries of the basin are marked by series of faults.

Since a general understanding of sedimentary basin features can be achieved through the interpretation of aeromagnetic anomaly data (Farhi et al., 2016; Mohamed et al., 2020), we have deployed geophysical data to provide further information needed to address the internal geometry of the basin. This is with the aim of locating structural features and estimating depths to magnetic sources by various processing techniques that have been established to aid the interpretation of magnetic data such as the estimation of source depth with the aid of source parameter imaging method (Thurston and Smith 1997). Furthermore, the application of first vertical derivative technique (Evjen, 1936) and total gradient method (MacLeod et al., 1993; Nabighian 1972; Roest et al., 1992) which are well adapted to locating structural features (intrusions, faults, contacts) have been deployed in this study.

In view of the discrepancy in estimated depth values of the Middle Niger Basin, we present the structural features and sedimentary thickness data of the Middle Niger Basin using high resolution aeromagnetic anomaly data. The objectives of the study are: **(i)** delineation of precise boundary of the basin using first vertical derivative, **(ii)** investigation of volcanic and structural features within the basin utilizing total gradient, **(iii)** possible depths to magnetic basement estimation using source parameter imaging technique. This study is aimed at providing important clues about the origin and internal geometry of the Middle Niger basin which are essential for further exploration activities within the basin.

## 2 Geologic setting

**Figure 2:**
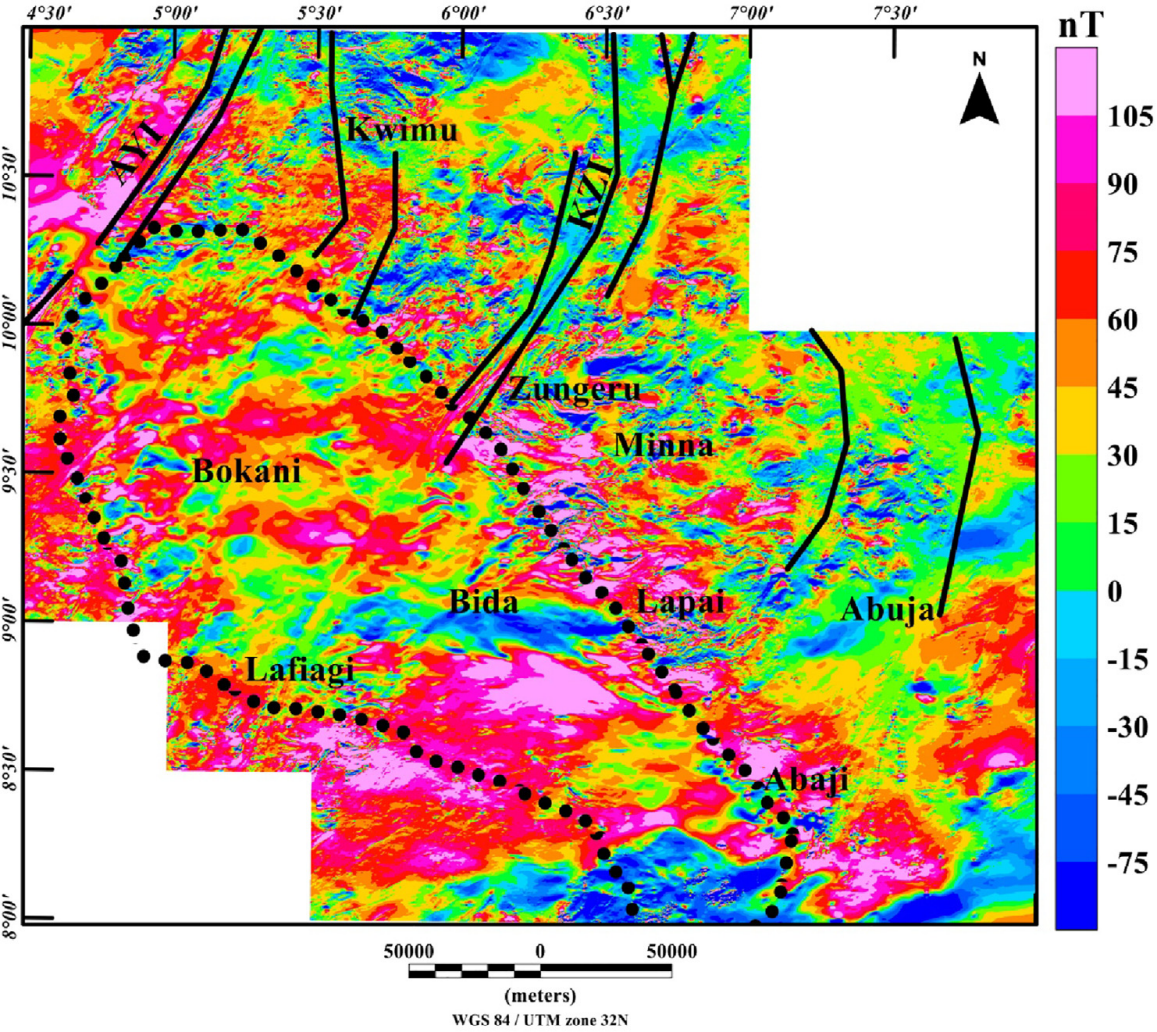
Total magnetic intensity anomaly map of the entire Middle Niger Basin and surrounding basement complex terrane. At low-latitude areas such as the studied region, magnetic source bodies are usually characterized by low aeromagnetic anomalies. The dotted lines represent the boundary of the Middle Niger Basin. Solid black lines: major shear zones from previous study of Salawu et al. (2020). Abbreviations: AYI – Anka-Yauri-Iseyin shear zone; KZI – Kalangai-Zungeru-Ifewara shear zone.

The studied area is located within 4° 30′ to 8° 00′ E and 8° 00′ to 11° 00′N in the central-western half of Nigeria (Figure 1). The Middle Niger basin (Figure 2) is presumed to be a north-westerly prolongation of the Anambra Basin (Akande et al., 2005). The sedimentary fill of the basin consists of a NW-trending belt of Upper Cretaceous sedimentary rock units which were deposited due to faulting of blocks, basement disintegration and subsidence consequent to the Cretaceous opening of the South Atlantic Ocean. The northern segment is commonly called the Bida sub-basin while the southern segment is referred to as Lokoja sub-basin. The Bida Formation lies unconformably on the Basement Complex in the northern segment of the basin. The Lokoja Formation which is its equivalent in the southern segment also unconformably overlies the basement. The Bida and Lokoja Formations are immature mineralogically and texturally, and consist of massive, clast to matrix supported conglomerate, that fine upwards to conglomeratic-sandstone, medium grained sandstone, siltstone and subordinate-claystone (Ojo and Akande, 2013). Overlying the Lokoja Formation is the Patti Formation which is made up of shale, sandstone, ironstone and claystone (Ojo et al., 2020). In the northern portion of the basin, the Bida Formation is overlain by the Enagi Formation which consists of siltstone, sandstone and claystone (Ojo 2012). The Enagi and Patti Formations are directly overlain by the Batati and Agbaja Formations respectively (Adeleye, 1974; Akande et al., 2005). The Batati Formation is made up of oolitic, goethitic and argillaceous ironstones with ferruginous siltstone and claystone intercalations and shaly beds occurring in small proportions, some of which yielded near-shore shallow marine to fresh water fauna (Adeleye, 1973). The Agbaja Formation (probably Late Maastrichtian) consists of oolitic and pisolitic ironstones (Ojo et al., 2020). The structural style of the Middle Niger Basin is portrayed by a system of NW-SE trending faults at the boundaries of the basin with the surrounding crystalline basement terrain (Kogbe et al., 1983; Rahaman et al., 2018), which suggests a rift origin for the Middle Niger Basin. No prominent morphological features, lineaments or intrusions were observed within the sedimentary basin (Salawu et al., 2020). In contrast, the underlying basement is characterized by prominent structural features, including the lateral continuity of the NNE-SSW trending Kalangai–Zungeru-Ifewara shear zones formed during the Pan-African orogeny (Salawu et al., 2020).

## 3 Data and methodology

The magnetic data investigated in this study was provided by the Nigerian Geological Survey Agency (NGSA). The high resolution aeromagnetic data is not publicly available but can be acquired from the NGSA. The data is part of the regional aeromagnetic data of Nigeria acquired by a geophysical company (Fugro Airborne Surveys Limited) for the NGSA between 2004 and 2009. The datasets were acquired at a constant flight altitude of 80 m on a series of NW-SE flight lines (perpendicular to regional trends). The flight lines are spaced and tied at 500 m and 2000 m respectively. The data were corrected for diurnal variation effects and the main component of the geomagnetic field (IGRF) was removed. A range of standard digital processing techniques were utilized for the interpretation of the aeromagnetic anomaly data to investigate the subsurface structural fabric of the Middle Niger Basin. These methods are commonly used for the interpretation of potential field dataset for locations and depth estimations of structural features such as faults, contacts and intrusive bodies. All processing of the aeromagnetic anomaly data was accomplished using the Oasis Montaj (Geosoft™) software package.

**Figure 3:**
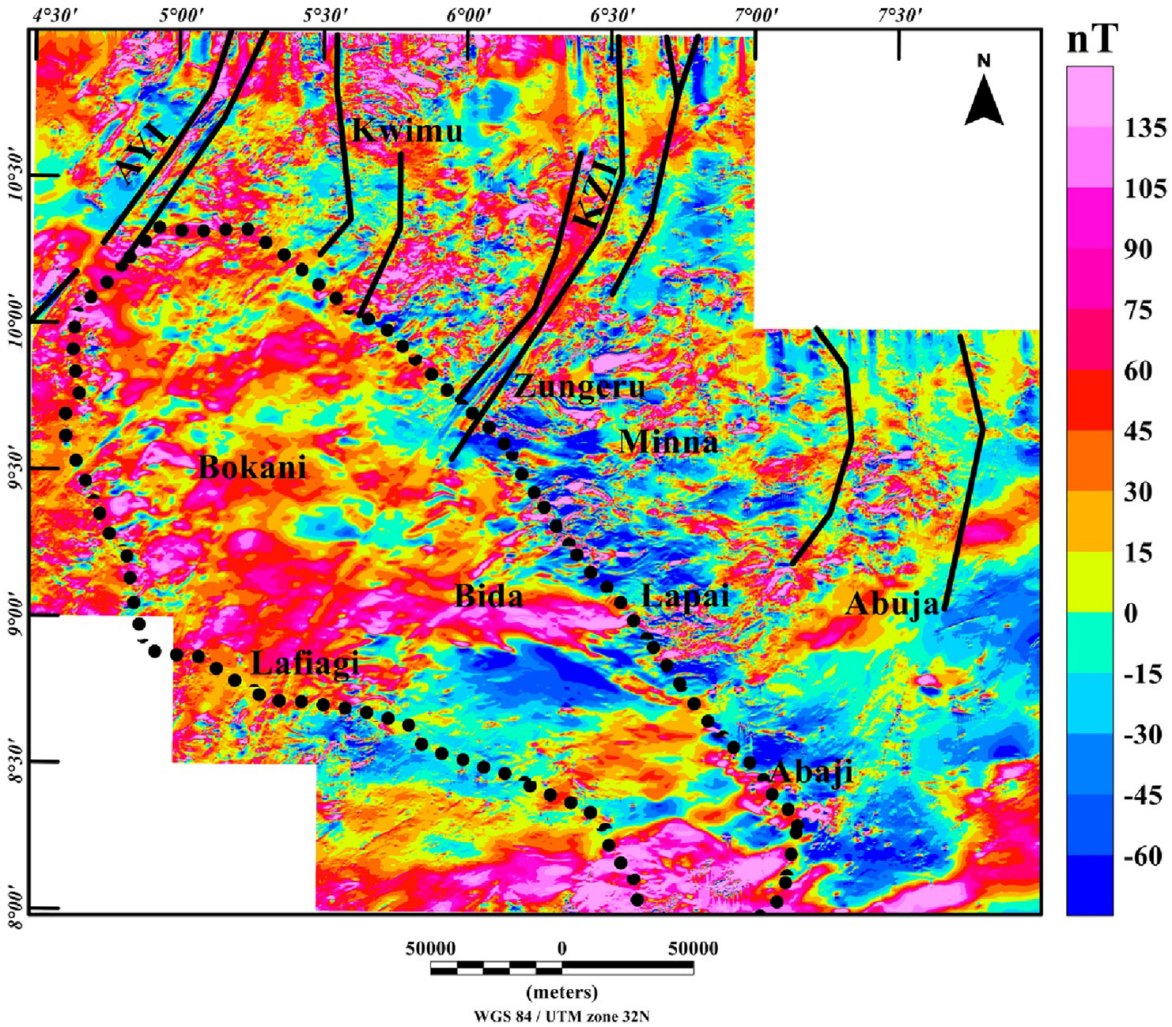
Reduced-to-pole aeromagnetic anomaly map of the Middle Niger Basin with surrounding basement complex terrane. The continuous dotted lines represent the boundary of the Middle Niger Basin. Solid black lines: major shear zones from previous study of Salawu et al., (2020). Abbreviations: AYI – Anka-Yauri-Iseyin shear zone; KZI – Kalangai-Zungeru-Ifewara shear zone.

Processing routine of the magnetic data comprised of the following steps: (a) the total magnetic intensity (TMI) anomaly data (Figure 2) was transformed to reduced-to-pole (RTP) data (Figure 3), to simplify the interpretation of magnetic anomalies which are influenced by the directions of the magnetic field and source magnetization (Saibi et al. 2015; Abdel Zaher et al., 2017). This transformation was achieved with an inclination: -4.978° and declination: -2.082 degrees of the Earth's field. The magnetic field parameters (inclination and declination) were chosen at the midpoint of the studied region (longitude: 6° 00′ E and latitude: 9° 30′N). Additionally, we implemented a pseudo-inclination 80° to stabilize the results of this filter, which is unstable in low-latitude regions. Considering the rock types and ages within the region, remanent magnetization is generally not an interfering factor, which allows the accurate use of the RTP transformation to correctly shift anomalies directly above their sources, (b) the application of first vertical derivative filter to enhance high frequency anomalies, in order to suppress the regional effects (long-wavelength) of deep magnetic sources. Hence, we examine the magnetic patterns of shallow source bodies, (c) application of total gradient technique to improve the magnetic signature of structures (faults or dykes), (d) calculation of depths to the magnetic basement using source parameter imaging technique for the tectonic framework. The enhancement filters and depth estimation technique utilized in this study are discussed below.

### 3.1 First vertical derivative

The first vertical derivative (FVD) technique is normally applied to aeromagnetic data to accentuate near surface structural features and shallow sources generating magnetic anomalies. The zero values of FVD of the reduced-to-pole (RTP) aeromagnetic field normally correspond to the geological structures. The FVD with respect to depth (z) is given in Eq. (1) as (Evjen, 1936):

(1) $$FVD=\frac{\partial F}{\partial z}$$

where F is the detected RTP aeromagnetic anomaly field. Here, we applied the FVD technique to the RTP aeromagnetic data to distinguish the Middle Niger Basin from the surrounding basement terrane based on the patterns of the FVD frequencies. Sedimentary basins are normally characterized by low anomaly values (high frequency) and the boundaries are marked by contrast of high and low anomaly values (Salawu et al., 2020).

### 3.2 Total gradient

The application of total gradient method to observed aeromagnetic anomaly field normally provides good horizontal locations for structures (such as contacts, intrusions, faults and sheet sources) independent of their magnetic latitude (Phillips 2000; Azizi et al., 2015). The amplitude of total gradient exhibits maxima over structural features, regardless of source magnetization directions and ambient magnetic field (Roest et al., 1992). The absence of the magnetization direction in the shape of the total gradient amplitude is an attractive characteristic for the analysis of aeromagnetic data measured around magnetic equator (MacLeod et al., 1993). The total gradient is derived from the three orthogonal gradients of the magnetic anomaly field using Eq. (2) (Roest et al. 1992), which is given as:

(2) $$TG\left(x,y\right)=\sqrt{{\left(\frac{\partial F}{\partial x}\right)}^{2}+{\left(\frac{\partial F}{\partial y}\right)}^{2}+{\left(\frac{\partial F}{\partial z}\right)}^{2}}$$

where TG(x,y) is the total gradient of the aeromagnetic anomaly field. The total gradient filter was applied to the aeromagnetic anomaly data of the study area to assess the existence of volcanic rocks in the Middle Niger Basin and to map structural features within the surrounding basement terrane.

### 3.3 Source parameter imaging

Interpretation of aeromagnetic anomalies requires determining each of the entity that characterized the sources of the magnetic anomalies. The source parameter imaging (SPI) is a major technique commonly used for the estimation of depth values of sources, which is an entity that is mostly sought (Smith et al., 1998). The estimated depth values are independent of magnetic declination, inclination, remanent magnetization, strike and dip (Thurston and Smith 1997). These characteristics make the SPI technique very useful for aeromagnetic anomaly data measured at low magnetic latitude regions such as the studied region. The SPI technique uses the local wavenumber in Eq. (3) for depth estimation which is given as:

(3) $$K=\frac{\frac{\partial^{2}F}{\partial x\partial z}\frac{\partial F}{\partial x}+\frac{\partial^{2}F}{\partial y\partial z}\frac{\partial F}{\partial y}+\frac{\partial^{2}F}{\partial z^{2}}\frac{\partial F}{\partial z}}{{\left(\frac{\partial F}{\partial x}\right)}^{2}+{\left(\frac{\partial F}{\partial y}\right)}^{2}+{\left(\frac{\partial F}{\partial z}\right)}^{2}}$$

SPI assumes a 2-D sloping contact model and maximum values of K are located above the sloping contact. In addition, depth values of magnetic sources are computed by substituting Eq. (3) into the expression for depth estimate in Eq. (4), given as:

(4) $$Depth_{x=0}=\frac{1}{K_{\max}}$$

where, K_max_ in Eq. (4) is the maximum value of K (equation 3) above 2-D sloping contact. The SPI technique was applied to the aeromagnetic data of the Middle Niger Basin and surrounding basement complex terrane to estimate depth values of sources within the basin and its surrounding areas. This is to obtain the sedimentary thickness of the basin and variations of depth values of sources within the surrounding basement terrane.

## 4 Result

### 4.1 Reduced-to-pole map

The Reduced-to-pole (RTP) map (Figure 3) shows magnetic high and low anomalies with different patterns within the study area. The RTP anomaly field ranges between −60 nT and 135 nT, revealing the high magnetic signature of the Middle Niger Basin and the surrounding basement complex areas. The RTP map can be classified generally into two main domains; the first domain which is characterized by scattered, high frequency magnetic anomalies correspond to the basement complex terranes of Kwimu, Zungeru, Minna, Lapai, and south of Lafiagi. The high frequency with low to moderate amplitudes anomalies within the basement terrane are interpreted as linked to shallow structures such as contacts, fractures and intrusive bodies. The second domain which reveals low frequency of high amplitude magnetic anomalies with prominent large elongated patterns corresponds to the Middle Niger Basin on the RTP map. This pattern of low frequency anomalies are attributed to deep-seated magnetic sources within the basement.

### 4.2 Structural features of the Middle Niger Basin and its basement

#### 4.2.1 First vertical derivative

**Figure 4:**
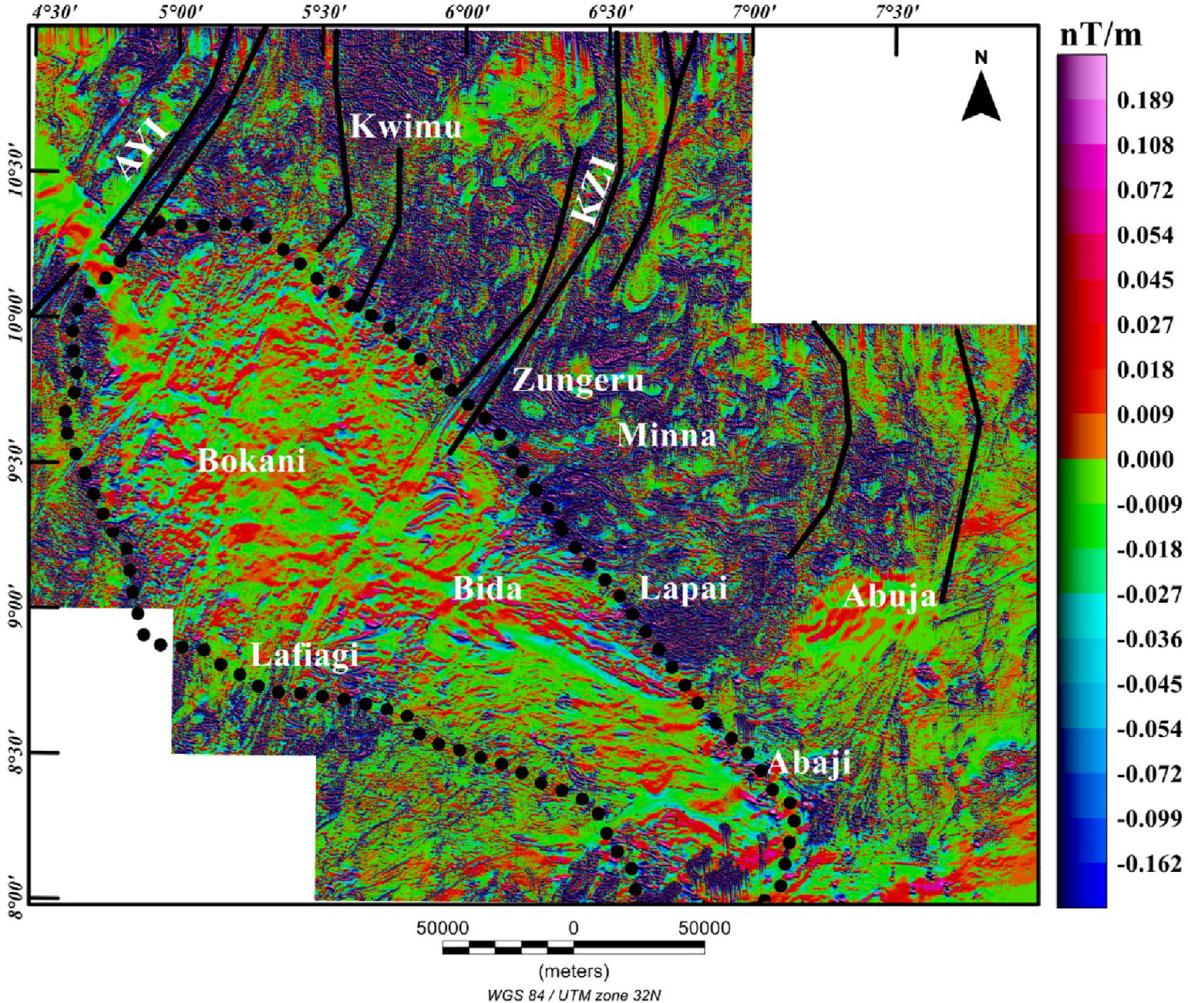
Color-shaded first vertical derivative (FVD) map produced from the reduced-to-pole aeromagnetic anomaly data of Middle Niger Basin and the surrounding basement complex terrane. The FVD enhances details of near surface sources and is suitable for delineating the edges and shape of sources. In the northeast and northern zones, high frequency pattern is seen in the basement complex terrane. The continuous dotted lines represent the boundary of the Middle Niger Basin. Solid black lines: major shear zones from previous study of Salawu et al., (2020). Abbreviations: AYI – Anka-Yauri-Iseyin shear zone; KZI – Kalangai-Zungeru-Ifewara shear zone.

The reduced-to-pole first vertical derivative (FVD) map (Figure 4) reveals rough relief, with negative and positive FVD anomalies of various amplitudes that ranges from −0.162 to 0.189 nT/m. The main magnetic trends are in the, NW-SE, N–S, NE–SW and E-W orientation within the basin and surrounding crystalline basement complex. In the basement terrane, elongated regional gradients with NNE–SSW trends are related to the Kalangai-Zungeru-Ifewara (KZI) shear zone around Zungeru area and Anka–Yauri-Iseyin (AYI) shear zone to the west. The low amplitude magnetic anomalies inside the basin, which extend from Bokani through Bida to Abaji areas are produced by a series of magnetic minerals of the Campano-Maastrichtian ironstones within the sedimentary formation that has been previously discussed, by Ojo and Akande (2013) and Ojo et al., (2020). This reveals possible lack of volcanic rocks within the sedimentary layers of the basin. These low amplitude magnetic anomalies are elongated and trends mainly in the NW-SE and E-W directions. A few elongated low amplitude magnetic anomalies with NE–SW trends are revealed within the central parts of the basin on the FVD map. Ojo and Akande (2013) have presented a detailed study of the southern portion of the Middle Niger Basin, and their results show heavy metal mineral assemblages such as magnetite, hematite, limonite and ilmenite within the Lokoja Formation of the basin. Generally, the FVD map reveals magnetic signature produced by shallow sources of the Middle Niger Basin and its surrounding crystalline basement areas.

#### 4.2.2 Total gradient

**Figure 5:**
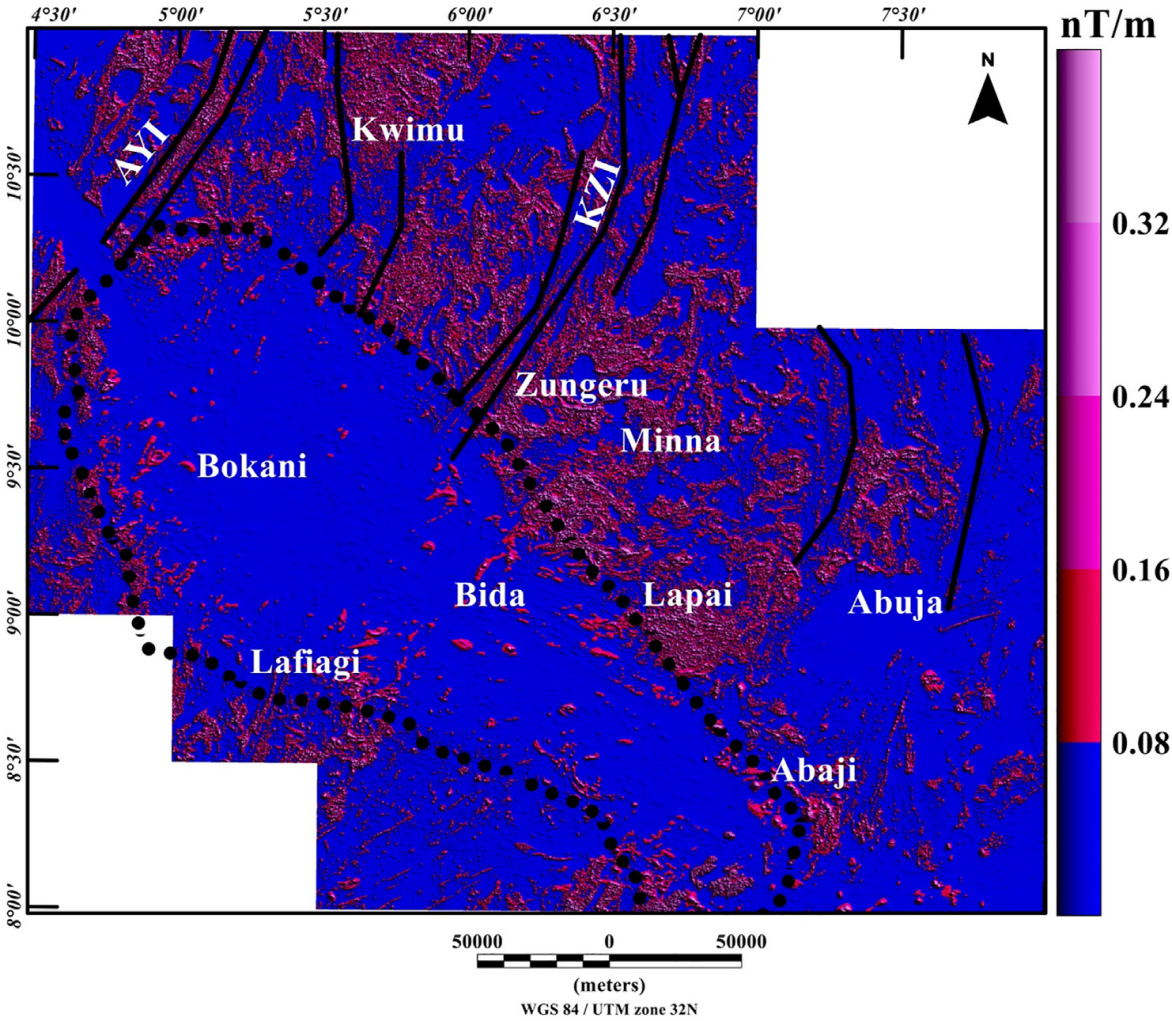
Color-shaded total gradient map of Middle Niger Basin and its surrounding crystalline basement terrane, computed from the aeromagnetic anomaly data. The continuous dotted lines represent the boundary of the Middle Niger Basin. Solid black lines: major shear zones from previous study of Salawu et al., (2020). Abbreviations: AYI – Anka-Yauri-Iseyin shear zone; KZI – Kalangai-Zungeru-Ifewara shear zone.

**Figure 6:**
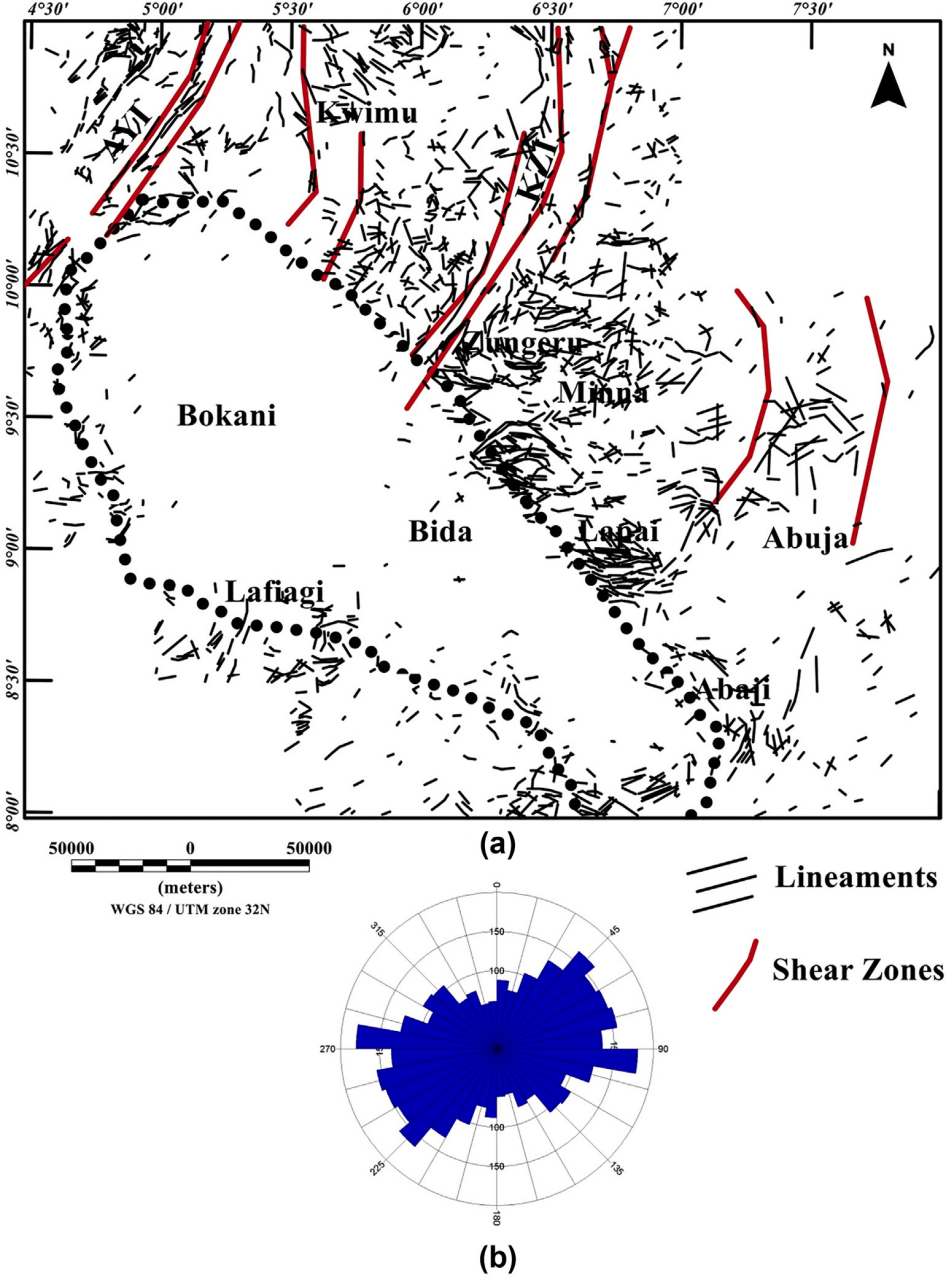
(a) Structural map of the study area produced from the extracted maxima of the total gradient amplitude, which was used for the identification of magnetic lineaments locations. (b) Rose diagram generated from the maxima of the total gradient amplitude reveals that lineaments are majorly oriented in the E-W, NE-SW and ENE-WSW directions. The continuous dotted lines on Figure 6a represent the boundary of the Middle Niger Basin. Solid red lines on Figure 6a are major shear zones from previous study of Salawu et al., (2020). Abbreviations: AYI – Anka-Yauri-Iseyin shear zone; KZI – Kalangai-Zungeru-Ifewara shear zone.

The total gradient map (Figure 5) shows negative and positive anomalies of various amplitude and wavelengths ranging from 0.08 to 0.32 nT/m (Figure 5). Inside the Middle Niger Basin (which extend from Bokani through Bida to Abaji areas), amplitude of the total gradient is minima which revealed a smooth surface on the total gradient map (see Figure 5). This denotes low magnetic contents of the Cretaceous sedimentary fill in the basin as well as probable non-volcanic rocks within the basin. The boundaries of the basement complex terrane with the sedimentary basin are marked by differences between low (0.08 nT/m) and high (0.32 nT/m) values of the total gradient amplitude, which are tentatively interpreted as series of faults. Dominating maxima of the total gradient amplitude becomes widely distributed towards the north and northeastern portion of the map (Figure 5) signifying an intensively fractured rocks within the basement complex terrane. In order to highlight the locations, lateral extent, and trends of structural features shown on the map, the maxima of the total gradient amplitude were extracted and used to produce the structural map (Figure 6) of the region under study. The visual inspection of the map (Figure 6) illustrates numerous magnetic structures which revealed the superficial shapes of the Middle Niger Basin. It also shows that the boundary of the basin is marked by systems of E-W, NW-SE and NE-SW trending lineaments. These trend patterns reveal the structural control architecture of the basin, thereby indicating a rift origin of the Middle Niger Basin. Additionally, the produced Rose diagram from the maxima of the total gradient amplitude reveals major orientation of lineaments in the E-W, NE-SW and ENE-WSW directions.

#### 4.2.3 Source parameter imaging

**Figure 7:**
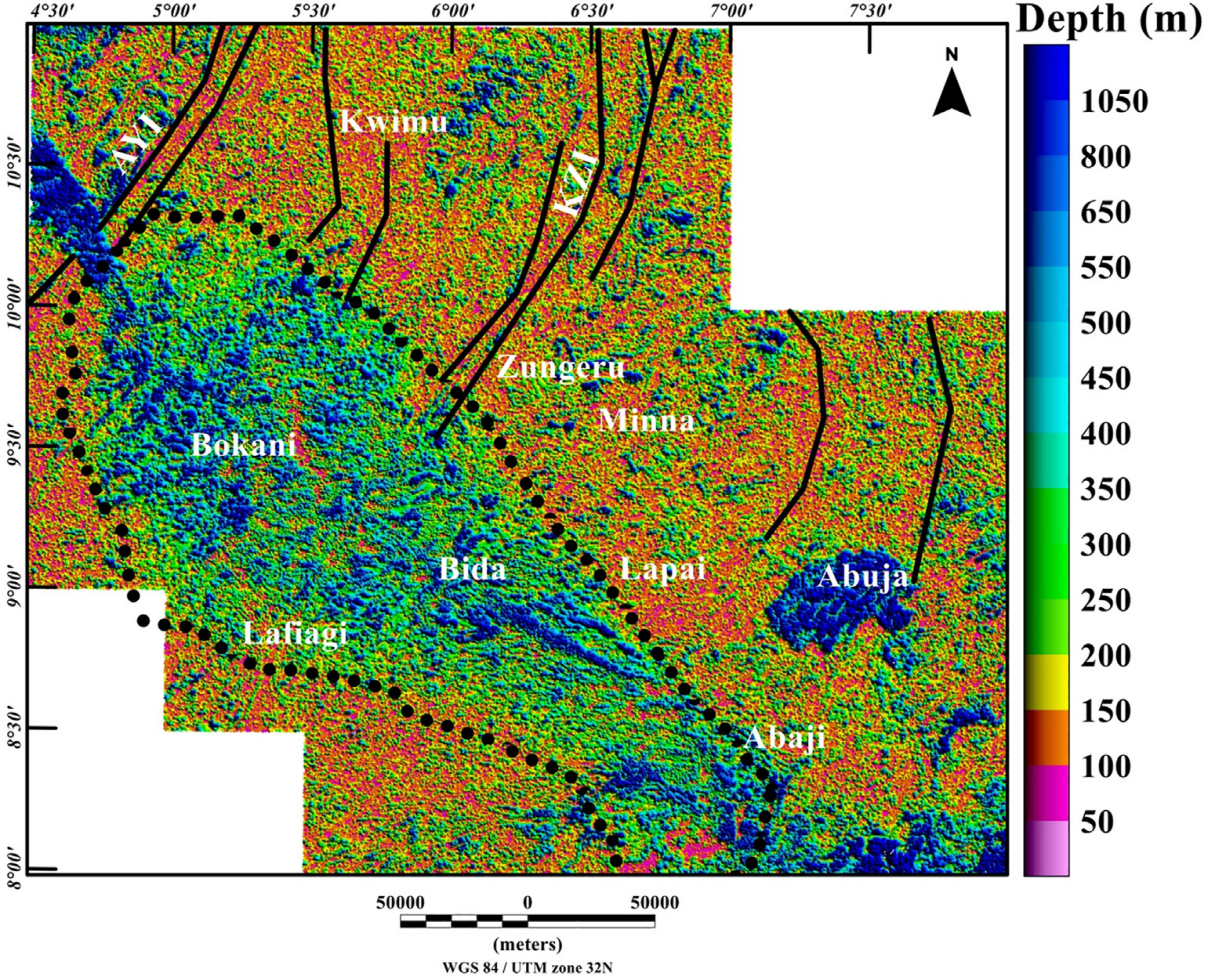
Color-shaded source parameter imaging depth map of Middle Niger Basin with surrounding basement areas derived from the total aeromagnetic data of the study area. The continuous dotted lines represent the boundary of the Middle Niger Basin. Solid black lines: major shear zones from previous study of Salawu et al., (2020). Abbreviations: AYI – Anka-Yauri-Iseyin shear zone; KZI – Kalangai-Zungeru-Ifewara shear zone.

**Figure 8:**
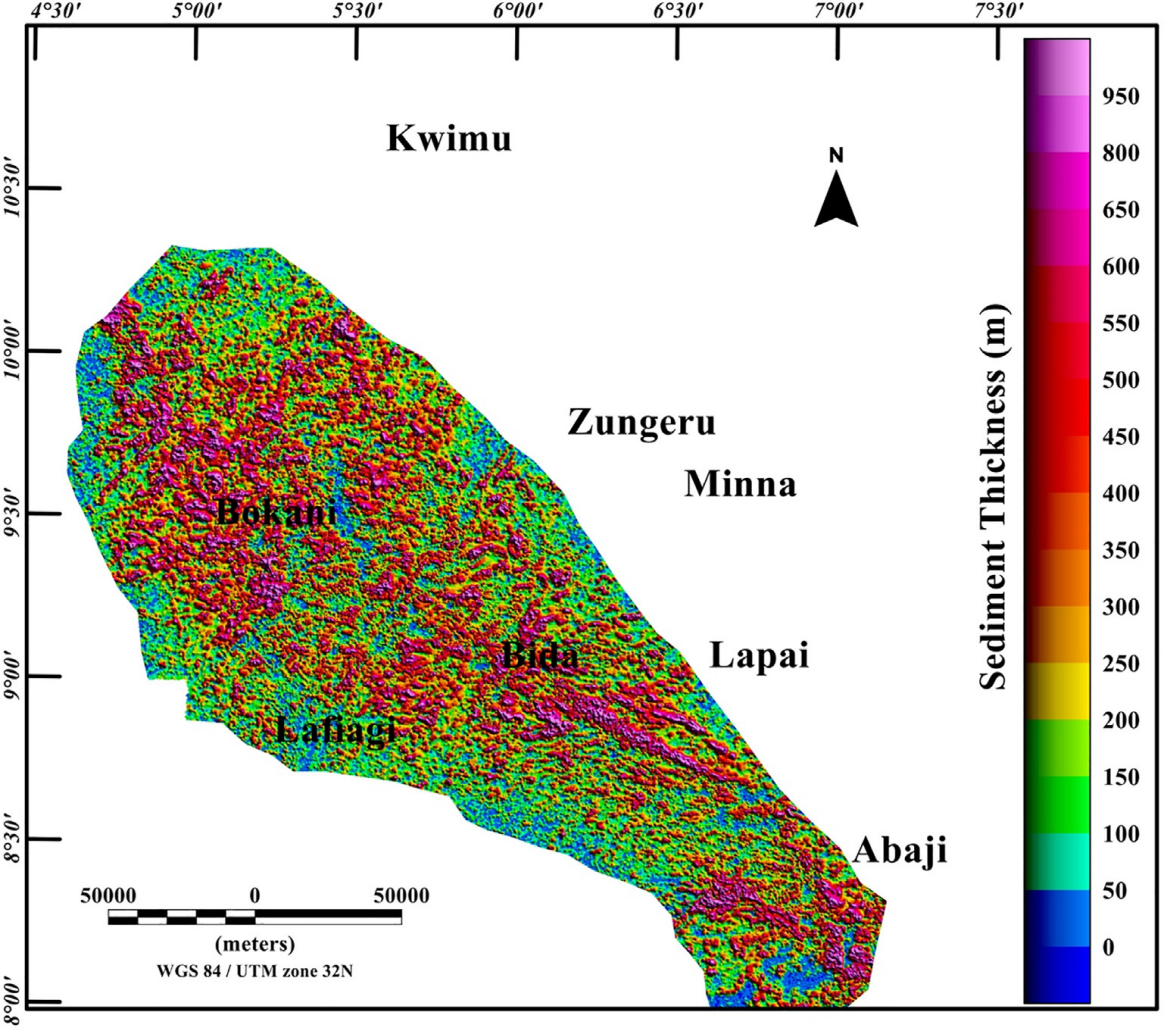
Color-shaded sedimentary thickness map estimated from the source parameter imaging depth map of Middle Niger Basin with surrounding basement areas.

The Middle Niger Basin is underlain and surrounded by the Nigerian Basement that carries imprints of the Pan-African tectonic event (600 Ma), with dominant N–S and NE-SW shears that define the major structural fabrics (foliations, lineations, folds, etc.) of the entire region (Dada 2008). One method which estimates the depth to the top of sources (such as sloping contact and thin sheet model) is by the application of the source parameter imaging (SPI) technique. These estimates can be used to represent the basin floor morphology, which has direct significance to the structural history of the region. The SPI map (Figure 7) reveals estimated depth values of sources within the studied region between 50 m and 1050 m. The depth values that relate to penetrative structural features within the crystalline basement complex terrane are mostly <150 m. A major fascinating feature on the map is the higher and consistent depth values of the entire Middle Niger Basin, which extends from Bokani through Bida to Abaji areas, in contrast to the lower depth values of the surrounding basement complex areas. The pattern of depth values in Figure 7 permits the visual assessment of the SPI map with the delineated boundaries of the Middle Niger Basin (revealed by the termination of structural features) on the structural map (Figure 6). The consistent depth values <1100 m depict the internal geometry of the Middle Niger Basin. Additionally, the extent of the Middle Niger Basin was extracted from the SPI Map to isolate the basin from the surrounding basement terrane. The value of 80 m sensor mean terrain clearance was subtracted from the resulting Middle Niger Basin SPI map to establish a sedimentary thickness map (Figure 8) of the basin. The map represents the estimated thickness of non-magnetic geological material above the magnetic basement.

## 5 Discussion

From recent studies that have added to our understanding of the origin of sedimentary basins resulting from structural controls (Ziegler and Cloetingh 2004; Lopes de Castro et al., 2012), we have sought to decipher the link between the underlying structural framework of the crystalline basement and the development of the Middle Niger basin. The interpretation of aeromagnetic data in this study has identified important structural features associated with geologic formations in the study area. It was observed that the reduced-to-pole (RTP) magnetic map (Figure 3) shows prominent high amplitude long wavelength RTP anomalies in Middle Niger Basin (Bokani and Bida areas) which are tentatively interpreted to be caused by magnetic sources within the basement that underlies the basin. These interpretations agree with the results of aeromagnetic investigation across various section of the basin by researchers such as Salawu et al., (2020) and Ojo (1990). The first vertical derivative (FVD) map of the RTP data was produced to focus on investigating shallow sources within the basin. The map reveals a clear contrast between the basin and surrounding crystalline basement. On the FVD map (Figure 4) many low amplitude magnetic anomalies are apparent inside the Middle Niger Basin (extending from Bokani through Bida to Abaji areas). Based on the magnitudes and narrow FVD anomalies, many of these appear to result from contrasts within the Cretaceous sedimentary formation of the basin. This is because anomalies produced by non-igneous magnetic sources within sedimentary formations are normally much weaker compared to those of the intrusive igneous rocks within the basement, which commonly contains a higher concentration of magnetic minerals (Gunn 1997). Additionally, the lack of maximum amplitude values of FVD anomalies inside the sedimentary layer of the Middle Niger Basin reveals a possible lack of volcanic rocks in the basin. In contrast, the basement complex shows several high amplitude anomalies attributed to shallow structural features such as intrusions, faults and fractures. The FVD map and its interpretation satisfy the results of the previous aeromagnetic investigation (Salawu et al., 2020; Ojo 1990) of the Middle Niger Basin, which suggests that the basin sedimentary pile is characterized by the absence of non-igneous intrusions. The total gradient map (Figure 5) reveals the proliferation of structures such as faults, folds and intrusions throughout the crystalline basement. The map equally shows the Middle Niger Basin to have fewer structures especially revealing the possible lack of volcanic rocks within the sedimentary layers of the basin. The produced structural map (Figure 6a) reveals basement fabrics and the superficial shapes of the Middle Niger Basin which is marked by E-W, NW-SE and NE-SW structural trends. The clouds of SPI depth solutions with NW-SE trends reveal the internal basin architecture. These trend patterns are orthogonal or oblique to the main basement fabric within the Middle Niger Basin, coupled with the presence of faults through the margin of the basin; thereby attributing its origin to a major rifting followed by sedimentation. The patterns of bounding lineaments of the Middle Niger Basin are parallel with basement fabrics. Hence, the bounding lineaments justify that the rift basin was controlled by the reactivation of pre-existing basement fabrics.

## 6 Conclusion

Aeromagnetic maps of the Middle Niger Basin and surrounding areas allow a comparison to be made between the structural signatures of the basin and its surrounding area. The presence of the shear zones and other magnetic lineaments within the crystalline basement complex terrane are outlined by prominent aeromagnetic lineaments. The first vertical derivative map depicts a possible lack of volcanic rocks within the Middle Niger Basin. The total gradient map revealed magnetic patterns, which allow the mapping of the basin boundaries. In contrast, the source parameter imaging map shows clouds of depth solutions, which suggest internal basin structures, trending mainly in the NW-SE direction perpendicular to the main basement fabric (NE-SE and E-W) deduced from the total gradient method. We use the source parameter imaging technique presented herein to establish magnetic depth estimates within the study area, though the magnetic basement topography varies considerably, the depths to the magnetic basement are generally not more than 1100 m.

## Declarations

### Author contribution statement

Naheem Banji Salawu, Silas Sunday Dada, Julius Ogunmola Fatoba, Olusola Johnson Ojo, Leke Sunday Adebiyi, Toyin Yusuf Abdulraheem: Analyzed and interpreted the data; Wrote the paper.

Abayomi John Sunday: Contributed reagents, materials, analysis tools or data.

### Funding statement

This research did not receive any specific grant from funding agencies in the public, commercial, or not-for-profit sectors.

### Data availability statement

The authors do not have permission to share data.

### Declaration of interests statement

The authors declare no conflict of interest.

### Additional information

No additional information is available for this paper.
